# A Psychoanalytically Informed Pilot Study of Moral Competence in German Young Adults Linked to Personality Structure and Parenting Experiences

**DOI:** 10.3390/bs16030341

**Published:** 2026-02-28

**Authors:** Aslı Akın, Holger von der Lippe, Jonathan Henssler, Inge Seiffge-Krenke, Stephan Doering, Stefan Gutwinski

**Affiliations:** 1 Department of Psychiatry and Psychotherapy, Charité-Universitätsmedizin Berlin, 10117 Berlin, Germany; asli.akin@charite.de (A.A.); jonathan.henssler@charite.de (J.H.); 2 Department of Psychology, Faculty of Sciences, MSB Medical School Berlin, 14197 Berlin, Germany; holger.vonderlippe@medicalschool-berlin.de; 3 Department of Psychology, University of Mainz, 55246 Mainz, Germany; seiffge-krenke@uni-mainz.de; 4 Department of Psychoanalysis and Psychotherapy, Medical University of Vienna, 1090 Vienna, Austria; stephan.doering@meduniwien.ac.at

**Keywords:** moral competence, personality structure, parenting experiences

## Abstract

The present pilot study explored associations between moral competence, personality structure and perceived parenting experiences. While previous research on moral competence has mainly emphasized educational and cognitive determinants, this work represents a novel psychoanalytically informed investigation of this ability. A sample of 88 young adults aged 18 to 21 completed an online survey including the *Moral Competence-Test*, *OPD-Structure-Questionnaire*, and *Zurich Brief Questionnaire for the Assessment of Parental Behaviors*. Exploratory analyses revealed a positive association between moral competence and overall integration of personality structure. Perceived parenting behaviors showed observable relationships with both constructs: warm and supportive parenting was associated with higher structural integration and greater moral competence, whereas parental control, particularly psychological control, was linked to lower personality structural and moral abilities. An exploratory mediation analysis further suggested that paternal warmth may indirectly affect moral competence via personality structure. This finding aligns with psychoanalytic theory proposing that father–child experiences, conceptualized as *triangulation*, create a cognitive and emotional space that fosters reflection and the development of moral competence. Overall, these exploratory findings underscore the need for longitudinal research examining the interplay between parenting experiences, personality structure, and moral development.

## 1. Introduction

In a world increasingly shaped by ethical complexity, digitalization, systemic crises, armed conflicts, and global interdependence, the capacity for young adults to engage in morally competent reasoning and behavior is of vital importance. *Moral competence*—understood as the ability to deliberate and resolve moral problems on the basis of universal principles through thinking and discussion rather than through violence, deceit, or power (Lind, 2024)—plays a central role in democratic engagement, social responsibility and peaceful conflict resolution. While considerable empirical attention has been devoted to moral-cognitive education within school and university settings, comparatively little research addresses the intrapsychic capacities that enable individuals not only to reason about moral dilemmas but also to emotionally tolerate, integrate, and act upon them.

From a psychodynamic perspective, moral functioning is largely rooted in the internalization of early relationship experiences with significant others, which form the *personality structure*—an evolving system of basic mental functions that becomes increasingly stable over time and operates largely outside conscious awareness (Rudolf, 2020). Traditionally, this perspective builds on Freud’s structural model of the psyche (Freud, 1923), in which the superego represents internalized parental norms and moral values acquired through early identification processes during childhood. Building on this framework, Kernberg (1975, 1984) was among the first to describe weaknesses in the superego as an impairment in personality structure. Contemporary psychodynamic theory further specifies these personality structural capacities as enabling realistic self- and other-perception, affect and impulse regulation, the expression of feelings and needs, and the formation of stable relationships (Task Force OPD, 2023). Deficits in these structural capacities have been empirically linked to reduced affect tolerance as well as increased reactive and appetitive aggression (Fritz et al., 2025; Kampe et al., 2025). Within this view, moral thinking, reasoning, and action emerge from the availability and integration of these underlying structural capacities. Investigating moral competence without attending to these psychodynamic foundations risks overlooking critical developmental pathways that operate beyond conscious awareness.

In contrast, classical developmental psychology (Piaget, 1973; Kohlberg, 1981) has predominantly conceptualized morality as a cognitive–developmental process evolving from externally guided obedience to autonomous judgment based on justice and universal ethical principles. While these stage models provided a valuable and still influential framework for understanding moral judgment in the context of social coexistence (e.g., Bulmash, 2024; Head, 2025; Jean-Tron et al., 2023; Martin et al., 2023; Moody-Adams, 2025), subsequent research questioned their assumption of a biologically determined moral progression (Turiel, 2024). Re-analyses of Kohlberg’s data showed that education, discourse climate, and social environment—rather than chronological age alone—play a crucial role in fostering moral reasoning (Lind, 1985, 2000). Based on these findings, Lind (2019) proposed the concept of moral competence as a measurable ability to apply moral principles consistently through reasoning and discussion, integrating both affective and cognitive dimensions. Numerous studies have empirically demonstrated the link between moral competence and prosocial behavior as well as democratic engagement (e.g., Blasi, 1980; Hemmerling, 2014; Lind, 2014; Lind et al., 2017; Miranda-Rodríguez et al., 2023; Presti et al., 2023).

Beyond educational and cognitive factors, early interactional experiences within the family have long been recognized as a central context for moral development. Parenting that combines warmth, responsiveness, and autonomy support fosters empathy, prosocial orientation, and advanced moral reasoning (Pastorelli et al., 2021; Pinquart & Fischer, 2022; X. Zhang et al., 2025), whereas authoritarian or emotionally neglectful parenting predicts moral disengagement and externalized moral control (Di Pentima et al., 2023; B. Wang & Liu, 2025; Y. Zhang et al., 2021). Yet, most of this research has remained on a behavioral or social-learning level, focusing on observable parenting styles rather than on how early interactional experiences with caregivers become internalized as enduring intrapsychic abilities or impairments that influence later moral functioning.

To integrate these findings with intrapsychic developmental processes, psychodynamic theory offers a framework for understanding how early relational experiences become internalized as enduring personality capacities. From a psychodynamic standpoint, such internalized early relational experiences shape expectations of emotional availability, trust, and reciprocity, which in turn guide how individuals perceive themselves and relate to others (Guntrip, 2018). These internalized patterns form the above-mentioned personality structure, which can be more or less integrated (Task Force OPD, 2023). Based on sufficiently good and fulfilling caregiving experiences, a well-integrated personality structure supports the capacity to reflect on one’s own motives and to consider others’ perspectives (Frank & Huber, 2021; Jauk & Ehrenthal, 2021)—functions that are essential for mature moral reasoning. Conversely, insecure or neglectful early relational experiences can lead to structural vulnerabilities, such as impaired affect regulation or limited perspective-taking (Fuchshuber et al., 2019; Fuchshuber et al., 2025), which in turn may constrain moral reflection and foster greater reliance on external authority or defensive moral disengagement.

Taken together, existing research demonstrates (a) that parenting practices are crucial for moral development, and (b) that a well-integrated personality structure provides the prerequisites for moral functioning by promoting the ability to self-reflect, take different perspectives, and regulate emotions. However, the dynamic interplay between these two levels—how early relationship experiences with parents shape moral competence through their impact on personality structure—has not yet been empirically examined. Addressing this gap, the present pilot study provides an initial psychoanalytically informed cross-sectional investigation of moral competence in young adults (18 to 21 years old), integrating measures of perceived parenting experiences and personality structure. This age range was selected because late adolescence and emerging adulthood represent a critical developmental period for the integration of personality structure and the consolidation of moral reasoning capacities (Arnett & Jensen, 2024; Eustice-Corwin et al., 2023). Based on psychodynamic theory and previous empirical findings, we hypothesized that: (H1) higher levels of personality structural integration would be positively associated with moral competence; (H2) perceived parenting characterized by high warmth/support and low psychological control would be positively associated with both personality structure and moral competence; (H3) the association between perceived parenting experiences and moral competence would be mediated by personality structure. By examining these interrelations, the study seeks to illuminate developmental pathways through which early relational experiences may become internalized as intrapsychic capacities that support moral competence, thus providing a foundation for future larger-scale research.

## 2. Materials and Methods

### 2.1. Data Collection and Study Design

This pilot study employed a cross-sectional online survey to investigate perceived parenting experiences, personality structure, and moral competence in young adults. Data were collected between April and July 2020. Recruitment took place in educational contexts in Germany through direct contact with teachers and university lecturers, who distributed the survey link to eligible students via classes or internal mailing lists. No public advertisements or social media postings were used, and participation therefore followed a convenience sampling approach within predefined settings rather than unrestricted self-enrollment.

Inclusion criteria were an age between 18 and 21 years and the provision of informed consent; no further exclusion criteria were applied. Participants were required to complete the core questionnaire sections on personality structure and moral competence to be included in the final analyses. The target sample size was determined a priori using G*Power 3.1.9.3, with a minimum of *N* ≥ 109 participants to achieve adequate statistical power.

Participants were informed about the study aims and data handling procedures, and provided informed consent prior to participation in accordance with local ethical guidelines and the General Data Protection Regulation (GDPR). Following recommendations by Lind (2019), the survey did not explicitly use the terms “moral” or “moral competence,” and no time limits were imposed.

### 2.2. Measures

**Figure A1:**
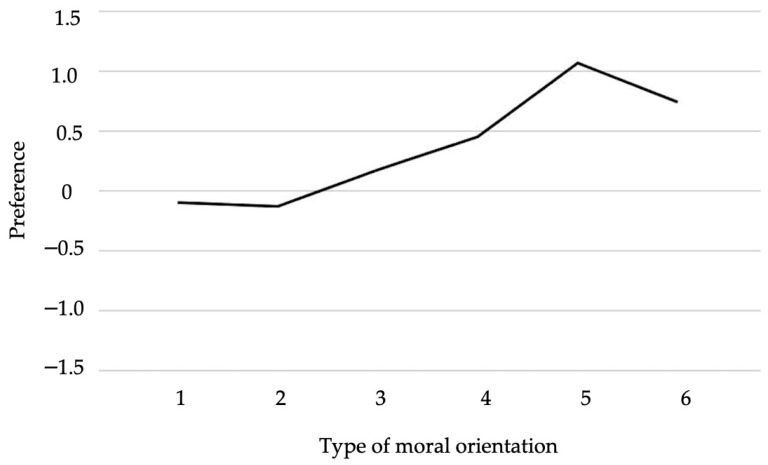
Average preference of the six types of moral orientation among the young adults surveyed (*N* = 88). The six types of moral orientation, based on Kohlberg’s theory, include: orientation toward punishment and obedience (type 1); instrumental and relativistic orientation (type 2); orientation toward interpersonal approval (type 3); orientation toward law and order (type 4); orientation toward the social contract (type 5); and orientation toward universal ethical principles (type 6).

**Figure A2:**
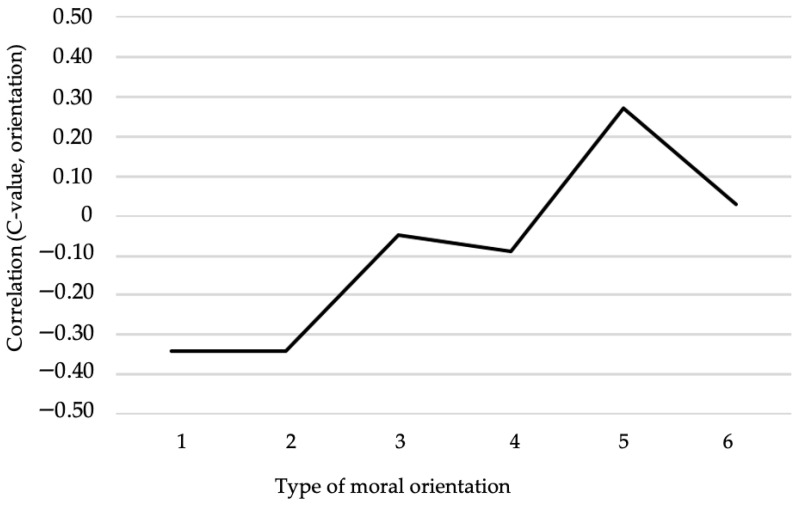
Affective-cognitive parallelism of moral abilities. The correlations between moral competence and moral orientations of the young adults surveyed are shown (*N* = 88).

*Moral Competence*. Moral competence was assessed using the *Moral Competence Test* (MCT; Lind, 1985), an experimental questionnaire designed to evaluate the effectiveness of specific dispositions in moral judgment (Lind, 2024). Participants were presented with two moral dilemmas: the “Worker Dilemma,” addressing workplace vigilantism, and the “Doctor Dilemma,” involving physician-assisted dying. For each dilemma, participants first rated the protagonist’s action on a scale from −3 (mostly wrong) to +3 (mostly right). They then evaluated six arguments supporting and six arguments opposing the behavior, reflecting different moral orientations derived from Kohlbergian theory, on a scale from −4 (*completely reject*) to +4 (*completely accept*). The MCT yields two scores: moral orientation (affective component) and moral competence (cognitive component, C-score). The C-score, ranging from 0 to 100, reflects the degree to which participants’ evaluations align with the moral quality of arguments rather than opinion conformity. The test’s validity is supported by the theoretical hierarchy of moral orientations, the affective-cognitive parallelism, and a quasi-simplex structure of intercorrelations (see Lind, 2019), all of which were confirmed in the present sample (Appendix A; Figure A1 and Figure A2; Table A1).

*Personality Structure.* The integration of personality structure was assessed via the *OPD-Structure Questionnaire* (OPD-SQ; Ehrenthal et al., 2012), a self-report instrument derived from the Operationalized Psychodynamic Diagnosis (OPD) system (Task Force OPD, 2023). The 95-item questionnaire captures the integration of the overall as well as eight sub-dimensions of personality structure (self-perception, object perception, self-regulation, regulation of relationships, internal communication, external communication, attachment to internal objects, attachment to external objects), rated on a 5-point Likert scale (0 = *not at all true* to 4 = *completely true*). Total scores can range from 0 to 380, with higher scores implying lower structural integration in terms of pathological polarity. To determine the overall integration of personality structure, the arithmetic mean of all items was calculated and then reversed to ensure a uniformly positive direction of the variables collected in the presented data analysis. Therefore, higher values in the following evaluations indicate a higher level of structural integration or availability of personality-structural abilities. The OPD-SQ demonstrates excellent internal consistency and construct validity, including strong associations with DSM-5 Level of Personality Functioning scores and differentiation between clinical and non-clinical samples (e.g., Bock et al., 2018; Doering, 2024; Ehrenthal et al., 2012; J. Zimmermann et al., 2015; Vierl et al., 2024). In line with this, psychometric analysis of the present data revealed acceptable to very good internal consistency for the sub-dimensions (Cronbach’s α values between 0.79 and 0.95) and a very high reliability for the overall scale (Cronbach’s α of 0.99).

*Parenting Experiences.* The participants’ parental experiences were measured using the *Zurich Brief Questionnaire for the Assessment of Parenting Behavior* (D-ZKE; Reitzle, 2015), assessing perceived parental behavior separately for mothers and fathers. In this context, *mother* and *father* refer to the primary female and male caregiving figures as perceived by the participant during upbringing, irrespective of biological relatedness. The 27-item version captures three parental dimensions from the perspective of the child/adolescent being raised: warmth/support, behavioral control, and psychological control, rated on a 4-point Likert scale (0 = *disagree* to 3 = *fully agree*). Psychometric investigations by Reitzle et al. (2024) demonstrated acceptable to good reliabilities for all subscales and confirmed the intended factor structure. Reliability analyses of the present sample showed good to excellent internal consistencies: warmth/support (α = 0.92 maternal, 0.96 paternal), behavioral control (α = 0.71 maternal, 0.86 paternal), and psychological control (α = 0.80 maternal, 0.91 paternal).

*Control Variables.* In addition to age and gender, perceived social support, educational status, and socioeconomic background were recorded. Perceived social support was measured using the German 5-item version of the *ENRICHD Social Support Instrument* (ESSI; Cordes et al., 2009), which demonstrated good psychometric properties in a recent large general population survey (Hinz et al., 2025) and accordingly high reliability in the present sample (α = 0.89). Parental socioeconomic status was assessed following the OECD’s Programme for International Student Assessment (PISA) framework (Avvisati & Wuyts, 2024), using the *International Socio-Economic Index* (ISEI) and parental years of education based on the *International Standard Classification of Education* (ISCED). For all analyses, the higher value of either parent was used.

### 2.3. Data Analysis

Data preparation and statistical analyses were conducted using IBM SPSS Statistics Version 29. The present pilot study aimed to explore associations between perceived parenting experiences, personality structure, and moral competence in young adults (18–21 years). Specifically, the analyses focused on the interrelationships between:

(1) personality structure and moral competence (H1),
(2) perceived parenting dimensions (warmth/support, behavioral control, psychological control) and both personality structure and moral competence (H2), and
(3) the mediating role of personality structure in the relationship between parenting experiences and moral competence (H3).

All analyses were conducted at a significance level of α ≤ 0.05. Given the exploratory nature of the study and the lack of prior empirical data, corrections for multiple testing were not applied (Boulesteix & Hoffmann, 2024). Participants outside the age range of 18–21 were excluded. Potential confounding variables, such as age, gender, perceived social support, educational status, and socioeconomic background, were examined in preliminary correlation and variance analyses. Social support was controlled for in analyses of personality structure and moral competence. The analytic strategy combined correlation analyses to assess associations and regression analyses to explore directed relationships implied by theory. Hierarchical regression was used for analyses directly derived from theoretical hypotheses, allowing us to control for potential confounding variables (e.g., social support) in a first step and to examine the incremental predictive value of personality structure or parenting dimensions in subsequent steps. Stepwise regression was applied in exploratory analyses to identify which specific parenting dimensions contributed most strongly to variance in personality structure and moral competence when multiple predictors were considered simultaneously, without imposing a predetermined order. Bootstrapped 95% confidence intervals (BCa, 5000 samples) were calculated for all correlation and regression coefficients. Effect sizes were interpreted according to Cohen (1988, 1992), whose conventions continue to be widely used in contemporary psychological and behavioral research (e.g., Lakens, 2022). Regression assumptions were examined and met following recommended guidelines (e.g., Savieri et al., 2025; Tabachnick & Fidell, 2013). Mediation was tested using PROCESS Version 3.5 (Hayes, 2018), with indirect effects considered significant if the 95% confidence interval did not include zero, in line with current methodological recommendations for bootstrap mediation analysis (e.g., Clement & Bradley-Garcia, 2022).

## 3. Results

### 3.1. Sample Characteristics

**Figure 1:**
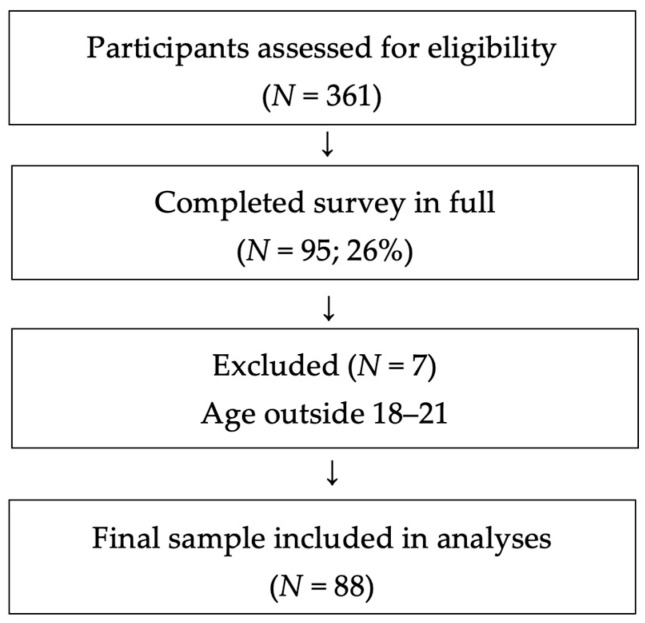
Flow-chart: Participant selection. *Note.* Authors’ own illustration.

Of the 361 individuals who accessed the survey, 95 (26%) completed all relevant items, while the remaining 266 participants were excluded due to incomplete responses. An exploratory comparison between completers and non-completers with regard to available demographic variables (age and gender) did not reveal substantial differences. Missing responses were more frequent in later sections of the survey. After excluding seven participants outside the target age range, the final sample comprised 88 participants. A flow-chart illustrating recruitment and exclusions is provided in Figure 1.

The sample had a mean age of 19.3 years (*SD* = 1.1) and comprised 57% female participants. Most were university students (36%) or high school students (34%), and lived in households of three to four members. The majority (76%) currently lived with their parents, and 84% had spent their childhoods living with both parents. Parental education was predominantly high school (mothers: 25%; fathers: 22%) or university level (mothers: 25%; fathers: 31%), and parents’ socioeconomic status ranged from very low (13%) to high (33%). See Table 1 for additional sociodemographic characteristics of the sample.

### 3.2. Descriptive Statistics

Descriptive statistics for the assessed variables are presented in Table 2. Participants’ scores ranged widely across measures of moral competence, personality structure, and perceived parenting dimensions.

### 3.3. Relationship Between Personality Structure and Moral Competence

Preliminary analyses indicated that control variables (gender, occupational status, educational attainment, parental socioeconomic status, and parents’ years of education) were not significantly associated with moral competence or personality structure (all *p* > 0.05). Perceived social support, however, showed a weak positive correlation with moral competence, *r*(86) = 0.22, *p* = 0.036, 95% CI [−0.03, 0.48], and a moderate, significant positive correlation with personality structure, *r*(86) = 0.41, *p* < 0.001, 95% CI [0.22, 0.58]. Consequently, social support was included as a covariate in subsequent analyses.

A partial correlation between moral competence and personality structure, controlling for social support, revealed a strong positive association, *r*(85) = 0.58, *p* < 0.001, 95% CI [0.42, 0.70].

Hierarchical regression analyses confirmed that personality structure significantly predicted moral competence, *F*(1, 86) = 49.50, *p* < 0.001, explaining 37% of the variance, *R*^2^ = 0.37, f^2^ = 0.59. Adding social support in a second step did not significantly improve model fit, *F*(2, 85) = 24.52, *p* < 0.001, nor increase explained variance (*R*^2^ = 0.37, f^2^ = 0.59). Personality structure remained a significant positive predictor (*b* = 10.13, *t*(86) = 7.04, *p* < 0.001; see Table 3).

### 3.4. Associations Between Perceived Parenting Experiences and Personality Structure

Control variables were not significantly associated with any parenting dimension (all *p* > 0.05). Correlation analyses between participants’ personality structure and their perceived parenting experiences showed that personality structure was moderately positively associated with maternal warmth/support, *r*(86) = 0.49, *p* < 0.001, 95% CI [0.32, 0.64], and strongly positively associated with paternal warmth/support, *r*(86) = 0.78, *p* < 0.001, 95% CI [0.67, 0.87]. Conversely, personality structure was negatively associated with maternal behavioral control, *r*(86) = −0.28, *p* = 0.004, 95% CI [−0.46, −0.09], and strongly negatively associated with paternal behavioral control, *r*(86) = −0.56, *p* < 0.001, 95% CI [−0.69, −0.42]. Strong negative associations were found between personality structure and maternal psychological control, *r*(86) = −0.48, *p* < 0.001, 95% CI [−0.62, −0.32], and paternal psychological control, *r*(86) = −0.79, *p* < 0.001, 95% CI [−0.86, −0.70]. All correlation coefficients are displayed in Table 4.

Stepwise regression showed that paternal psychological control alone explained 62% of the variance in personality structure, *R*^2^ = 0.62, f^2^ = 1.63, *F*(1, 86) = 139.67, *p* < 0.001. Adding paternal warmth/support increased explained variance to 68%, *R*^2^ = 0.68, f^2^ = 2.13, *F*(2, 85) = 88.68, *p* < 0.001. Paternal psychological control was a significant negative predictor (*b* = −0.46, *t*(85) = −4.13, *p* < 0.001), whereas paternal warmth/support was a significant positive predictor (*b* = 0.41, *t*(85) = 3.87, *p* < 0.001; Table 5). Maternal warmth/support, maternal psychological control, and parental behavioral control dimensions were not significant predictors.

### 3.5. Associations Between Perceived Parenting Experiences and Moral Competence

Pearson correlations indicated that maternal warmth/support was weakly positively associated with moral competence, *r*(86) = 0.28, *p* = 0.004, 95% CI [0.08, 0.46], and paternal warmth/support was moderately positively associated, *r*(86) = 0.49, *p* < 0.001, 95% CI [0.32, 0.64]. Maternal behavioral control was not significantly related, *r*(86) = −0.09, *p* = 0.215, whereas paternal behavioral control was moderately negatively associated, *r*(86) = −0.45, *p* < 0.001. Maternal psychological control showed a weak negative association, *r*(86) = −0.19, *p* = 0.038, and paternal psychological control a moderate negative association, *r*(86) = −0.48, *p* < 0.001.

Stepwise regression revealed that paternal warmth/support alone accounted for 24% of variance in moral competence, *R*^2^ = 0.24, f^2^ = 0.32, *F*(1, 86) = 27.44, *p* < 0.001. Adding paternal behavioral control increased explained variance to 30%, *R*^2^ = 0.30, f^2^ = 0.43, *F*(2, 85) = 17.90, *p* < 0.001. Paternal warmth/support was a significant positive predictor (*b* = 5.87, *t*(85) = 3.40, *p* < 0.001), whereas paternal behavioral control was a significant negative predictor (*b* = −4.57, *t*(85) = −2.57, *p* = 0.012; Table 6). Maternal dimensions and paternal psychological control were not significant predictors.

### 3.6. Mediation of the Effect of Paternal Warmth/Support on Moral Competence via Personality Structure

**Figure 2:**
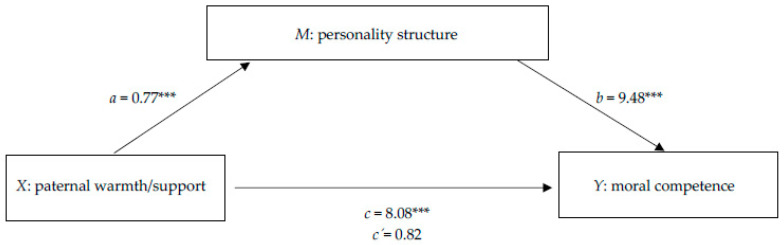
Mediation model predicting moral competence from perceived paternal warmth/support, considering the mediating effect of personality structure. Unstandardized path coefficients are presented. *R*^2^ = 0.37, f^2^ = 0.59. *** *p* < 0.001. *Note.* Authors’ own illustration.

Based on the regression analyses above, only paternal warmth/support significantly predicted both moral competence and personality structure. Mediation analysis confirmed that the effect of paternal warmth/support on moral competence was fully mediated by personality structure (Figure 2). Paternal warmth/support significantly predicted moral competence, *c* = 8.08, *p* < 0.001, 95% CI [5.02, 11.15]. When personality structure was included as a mediator, paternal warmth/support significantly predicted personality structure, *a* = 0.77, *p* < 0.001, 95% CI [0.64, 0.90], which in turn significantly predicted moral competence, *b* = 9.48, *p* < 0.001, 95% CI [4.86, 14.09]. The direct effect of paternal warmth/support on moral competence was no longer significant, *c′* = 0.82, *p* = 0.721, 95% CI [−3.71, 5.34], indicating full mediation. The indirect effect was significant, *ab* = 7.27, 95% CI [3.72, 11.28].

## 4. Discussion

This study investigated, from a psychoanalytically informed perspective, how perceived parenting experiences and the integration of personality structure are related to moral competence in early adulthood. The findings indicate that personality structure mediates the relationship between perceived paternal warmth/support and moral competence, highlighting the potential importance of fathers in both structural and moral development. Below, we discuss the main findings, limitations and implications for future research.

### 4.1. Personality Structure and Moral Competence

As expected, higher integration of personality structure was associated with higher moral competence, supporting psychoanalytic perspectives that personality structural capacities—such as affect regulation, impulse control, and self-reflection—facilitate coherent moral reasoning (e.g., Frank & Huber, 2021; Guntrip, 2018; Task Force OPD, 2023). Regression analyses suggest that personality structure provides a framework for reflective moral deliberation, consistent with Lind’s (2024) conceptualization of moral competence.

Although Gerd Rudolf—a leading German representative of structure-oriented psychodynamic psychotherapy—does not explicitly address the construct of moral competence, he emphasizes that ethical responsibility is rooted in the personality structure (Rudolf, 2018, 2020, 2023). To date, this assumption has not been directly examined empirically within Rudolf’s theoretical framework. However, so far different empirical investigations have been able to link impairments in personality structure with deficits in reflective functioning, perspective-taking, and interpersonal problems (e.g., Fritz et al., 2025; Fuchshuber et al., 2025; Jauk & Ehrenthal, 2021). These findings provide indirect empirical support for our results by suggesting that more integrated structural functioning may form the psychological foundation necessary for engaging in responsible, reflective moral reasoning.

However, due to the cross-sectional design and explorative nature of the analyses, causality cannot be inferred. While psychodynamic theory posits that personality structure develops through early relational experiences, it remains dynamic, and engaging with moral challenges in adulthood may further promote structural maturation. Longitudinal studies are needed to clarify these directional relationships.

### 4.2. Parenting Experiences and Personality Structure

Both maternal and paternal warmth/support were positively correlated with personality structure, but only paternal warmth/support predicted structural integration in regression analyses. This challenges traditional psychoanalytic emphasis on the mother as the primary attachment figure (e.g., Poscheschnik & Traxl, 2016; P. Zimmermann et al., 2022), suggesting fathers may play a distinct role in early adulthood/late adolescence. Contemporary father research supports this perspective, indicating that sensitive paternal involvement promotes emotion regulation, autonomy, and exploration, facilitating structural development (Seiffge-Krenke, 2024).

Perceived parental behavioral and psychological control—particularly paternal—was negatively associated with personality structure, consistent with evidence linking intrusive parenting to delayed identity development and socio-emotional problems (Seiffge-Krenke & Escher, 2017; Jiang et al., 2023). These findings underscore the potential risks of overcontrolling parenting during young adulthood, a period characterized by autonomy needs (Anderson, 2025; Seiffge-Krenke, 2023).

Notably, a high proportion of the variance in the personality structure of the participants could be explained by their perceived paternal psychological control. In conjunction with the high positive effect of perceived paternal warmth/support, this finding indicates the outstanding importance of fathers for personality structural abilities in early adulthood (Seiffge-Krenke, 2024). Although these associations cannot be interpreted causally, they underscore the potential risks of overcontrolling parenting on the structural development.

### 4.3. Parenting Experiences and Moral Competence

Parental warmth/support, particularly paternal, was positively associated with moral competence, whereas parental behavioral control showed no positive effects and even a negative predictive value in the case of paternal behavioral control. Again, strong parallels to the findings from Seiffge-Krenke’s (2016, 2024) developmental psychological research on the importance of fathers over the last decade become apparent. Our findings suggest that fathers’ warmth and involvement can predict moral competence more strongly than maternal contributions, possibly due to processes of identification and triangulation in young adulthood that facilitate reflective thinking. From a psychoanalytic perspective, triangulation experiences involving the father create a cognitive and emotional “space” for reflection and moral deliberation, supporting the development of autonomous thinking (Oliveira, 2024), which is a prerequisite for moral competence according to Lind (2024).

The negative association between parental behavioral and psychological control and moral competence can be understood in light of the conceptual distinction between moral competence and moral norms or ideals. Moral competence, unlike externally imposed rules and norms, requires reflection, discussion, and the consideration of alternative viewpoints (Lind, 2024). Excessive parental control, especially from fathers, may hinder these processes, limiting opportunities for autonomous moral reasoning.

As noted above, the cross-sectional data prevent causal conclusions; it remains unclear whether paternal warmth fosters moral competence or if morally competent young adults are more likely to perceive and recall warmth in the relationship to their fathers.

### 4.4. Mediating Role of Personality Structure

Mediation analyses showed that personality structure fully mediated the effect of paternal warmth/support on moral competence, suggesting that a warm and supportive father–child relationship fosters the development of more integrated structural capacities (e.g., emotional regulation, self-reflection), which in turn facilitate advanced moral reasoning. This supports psychoanalytic theory that fathers foster autonomy, reflective capacities, and moral reasoning through triangulation and relational engagement (Abelin, 1971; Berman & Long, 2022; Bion, 1962).

A recent contribution by Dacka (2025) further supports this interpretation. In her article *The Role of the Father in the Formation of a Child’s Moral Intelligence* she reviews literature suggesting that fathers play a crucial role in shaping moral intelligence by introducing children to moral values, encouraging prosocial behaviors, and engaging in meaningful moral conversations from early childhood onward. According to Dacka (2025), paternal involvement, relational closeness, and active participation in moral socialization provide a foundation for children to internalize moral principles, develop discernment, and form an ethical orientation that persists into later life.

Our findings complement Dacka’s theoretical claims in several ways. First, by demonstrating a statistical mediation, we provide empirical support to the idea that it is not merely the presence of the father or his moral instruction that matters, but how his warmth and support are internalized to build a structural capacity in the child—or, in our case, the young adult—that enables moral deliberation. Second, by focusing on moral competence (rather than only moral values or behavior), our data suggest that the integration of personality structure is the “mechanism” through which paternal warmth translates into complex moral judgment, not just moral conformity or internalized norms.

Beyond psychoanalytic theory, developmental and moral psychology research also backs the central role of parental warmth–especially paternal warmth–for moral development. For example, Hennig and Walker (1999) showed that parental interaction styles characterized by warmth, Socratic questioning, and support strongly predicted children’s moral reasoning growth over time. Walker and Taylor (1991) also found that parental discussion of moral conflicts, combined with higher-level reasoning, was particularly beneficial for children’s development of moral judgment. A more recent meta-analysis further supports this link, indicating that consistent parental warmth is positively associated with higher moral reasoning in children and adolescents (Pinquart & Fischer, 2022). These foundational studies resonate with our mediation finding: paternal warmth appears to be a relational ingredient that enables deeper cognitive and structural moral engagement.

Large-scale cross-cultural research further underscores the importance of paternal warmth. For instance, a longitudinal study across nine countries found that father-based effects (warmth more than control) on child adjustment were frequent and persistent, suggesting a robust and somewhat universal role of paternal warmth in emotional and behavioral development (Rothenberg et al., 2020). While that study focused on internalizing and externalizing symptoms rather than moral competence specifically, it supports the plausibility that paternal warmth has enduring, cross-domain effects, potentially including moral dimensions.

Beyond psychoanalytic and developmental perspectives, the present mediation finding can also be interpreted from a sociological standpoint, particularly through Symbolic Interactionism. From this lens, moral capacities develop through social interactions in which the self is continuously constructed and negotiated with others (Koudenburg et al., 2024). Warm and supportive parental interactions provide opportunities for perspective-taking, feedback, and internalization of norms (L. Wang, 2023), highlighting that moral competence is relationally shaped. Within this framework, personality structure may be understood as the psychological mechanism that enables individuals to translate social experiences–especially with fathers–into coherent moral reasoning. This complementary perspective broadens the theoretical interpretation of the mediation effect and suggests additional avenues for future research.

### 4.5. Limitations

Several methodological limitations should be considered when interpreting the findings. First, the sample size of *N* = 88 was relatively small, which limited the statistical power to detect effects of small magnitude (Lakens, 2022). Post-hoc sensitivity analyses indicated that only moderate to large effects could be reliably detected, suggesting that non-significant associations, for example between maternal parenting dimensions and personality structure or moral competence, should be interpreted cautiously. Accordingly, the stronger predictive role of paternal warmth observed in this study should be interpreted cautiously and not as indicating the absence of maternal influences. Future research would benefit from larger samples to allow detection of smaller but potentially meaningful effects.

Second, no corrections for multiple testing (e.g., Bonferroni adjustment) were applied. Given the exploratory and pilot nature of the study, this decision was made to avoid an overly conservative inflation of Type II error and the premature dismissal of potentially meaningful associations. However, this approach increases the risk of Type I errors, and some statistically significant findings may represent false positives (e.g., Barnett et al., 2022; Turolla et al., 2023). Accordingly, the results—particularly those from exploratory analyses—should be interpreted with caution and regarded as hypothesis-generating rather than confirmatory. Replication in larger, adequately powered samples with appropriate corrections for multiple testing is required.

Third, the cross-sectional design of the study prevents causal inferences. Although the mediation model assumes that parental behaviors precede the development of personality structure, which in turn supports moral competence, this temporal ordering cannot be empirically confirmed in the present dataset. Prior research has highlighted that child personality and perception of parenting can mutually influence each other (Milovanović et al., 2023; Krohne & Hock, 2015; Ratzke et al., 2003), underscoring the potential bidirectionality of these associations.

Fourth, all data were collected via self-report instruments from young adults, which limits generalizability. Retrospective perceptions of parenting may be influenced by current mood, cognitive biases, or the respondents’ own personality and moral development. Although prior studies suggest that adolescent reports of parental behavior can be predictive of developmental outcomes (Frick et al., 1999; Kapetanovic & Boson, 2022; Reitzle et al., 2001), the absence of parental or expert assessments constrains the objectivity of the measures.

Fifth, the sample consisted exclusively of young adults in a narrow developmental stage (late adolescents/emerging adulthood), predominantly from medium to high socioeconomic backgrounds, and included only individuals identifying as women or men. This limits the representativeness of the findings and their generalizability to other age groups, socioeconomic contexts, and individuals identifying outside the gender binary. In particular, the pronounced role of paternal warmth observed in this study should be interpreted as specific to this developmental and sociocultural context. For example, Buckley et al. (2024) showed that associations between parental warmth and psychosocial outcomes vary across developmental stages, while Bornstein et al. (2022) demonstrated that such associations are further shaped by socioeconomic and gender-related contexts, underscoring that the salience of parental influences is neither developmentally nor socially invariant. Accordingly, it cannot be assumed that similar patterns would emerge in younger adolescents, later adulthood, or populations facing different socioeconomic conditions. Against this background, future studies should investigate whether the relative contributions of maternal and paternal parenting vary across developmental stages, social contexts, and gender identities.

Finally, the study did not assess participants’ mental health status. Psychological symptoms such as depression, anxiety, or externalizing behaviors may influence both the perception of parental behavior (e.g., Florean et al., 2022) and performance on measures of personality structure (e.g., Kerber et al., 2024) or moral competence (e.g., Spilg et al., 2022). The absence of mental health covariates therefore limits the ability to disentangle whether observed associations reflect developmental processes or are partly attributable to unmeasured psychopathology. Future studies should incorporate standardized assessments of mental health to clarify these relationships.

### 4.6. Implications

The results of the presented pilot investigation have theoretical, clinical, and parenting-related implications. Theoretically, the present study represents the first attempt to examine moral competence from a psychodynamic perspective and to integrate it into socialization and developmental research. Consistent with our first hypothesis (H1), higher levels of personality structural integration were positively associated with moral competence, supporting the notion that well-integrated personality capacities—such as emotion regulation, impulse control, and self-reflection—facilitate advanced moral reasoning. In line with our second hypothesis (H2), perceived parenting characterized by warmth and support, particularly paternal, was positively associated with both personality structure and moral competence, whereas parental control—especially psychological control—showed negative associations with these outcomes. Finally, supporting our third hypothesis (H3), mediation analyses indicated that personality structure fully mediated the effect of perceived paternal warmth/support on moral competence, suggesting that relational experiences with fathers contribute to moral abilities through the facilitation of integrated structural capacities. While these findings must be interpreted in light of the study’s limitations, they underscore the potential significance of fathers in the structural and moral development of young adults.

Clinically, these findings suggest that interventions aimed at supporting the development of personality structure may indirectly enhance moral reasoning capacities. Parenting and family-based intervention programs may benefit from emphasizing the distinctive contributions of fathers, particularly through warm, supportive, and autonomy-promoting involvement. Such programs could help foster both structural integration and moral competence in adolescents and young adults.

From a developmental perspective, the present findings highlight the need for longitudinal research tracking both personality structural and moral development in relation to parenting behaviors from early childhood through adulthood. Longitudinal studies could clarify the age-dependent impact of paternal involvement and explore how specific dimensions of personality structure contribute to moral competence, allowing for a more nuanced understanding of the interplay between structural and moral capacities.

Overall, further research on personality structural and moral development in young adults from a psychodynamic standpoint is encouraged. Identifying parenting practices that promote both personality structural and moral growth may contribute not only to individual maturation but also to the development of collective skills in conflict resolution and democratic participation. Although the present study cannot definitively answer the question of how to optimally foster moral competence through early relational experiences, it provides promising avenues for interventions that complement existing education-focused approaches. In sum, these findings suggest a novel perspective for moral development research: moral capacities in young adults appear closely linked to foundational personality functioning, with warmth and support—particularly perceived from fathers—serving as a key environmental contributor.

## Figures and Tables

**Table 1:** Sociodemographic characteristics of the sample (*N* = 88).

Characteristic	*n*	%
*Gender*		
female	50	57
male	38	43
*Age*		
18	26	30
19	24	27
20	21	24
21	17	19
*Participants’ highest educational qualification*		
secondary school diploma	20	23
vocational training/trade school	2	2
high school/university entrance qualification	57	65
university degree (BA/MA/BSc/MSc)	9	10
*Parental socioeconomic status* ^a^		
very low	11	13
low	17	19
medium	25	28
high	29	33
not codable	6	7

*Note.* Table structure and categories reproduced from Akın (2020, p. 30). Original German labels and descriptions were translated into English. ^a^ Based on the Highest International Socio-Economic Index of Occupational Status (HISEI; Avvisati & Wuyts, 2024) and classified according to Lohmann et al. (2009). The category *not codable* was used for responses that could not be reliably assigned to a coding category due to ambiguity or insufficient information.

**Table 2:** Descriptive statistics of study variables.

Variable	*Min*	*Max*	*M*	*SD*
Moral competence	0.53	72.33	17.6	15.0
Personality structure	0.61	3.88	2.1	0.9
Maternal warmth/support	0.00	2.92	1.9	0.7
Paternal warmth/support	0.00	3.00	1.4	0.9
Maternal behavioral control	0.67	3.00	1.8	0.6
Paternal behavioral control	0.00	3.00	1.7	0.9
Maternal psychological control	0.00	2.44	1.0	0.6
Paternal psychological control	0.00	3.00	1.3	0.9

*Note. N* = 88. *Min* = minimum; *Max* = maximum.

**Table 3:** Hierarchical regression predicting moral competence from personality structure.

Model	Variable	*b*	95% CI	*SE_b_*	β	*t*	*p*	*R* ^2^
1	Constant	−3.60	[−10.10, 2.89]	3.27	—	−1.10	0.273	0.37 ***
	Personality structure	10.13	[7.27, 12.99]	1.44	0.60	7.04	<0.001	
2	Constant	−2.20	[−14.25, 9.86]	6.06	—	−0.36	0.718	0.37 ***
	Personality structure	10.31	[7.16, 13.45]	1.58	0.62	6.51	<0.001	
	Social support	−0.10	[−0.78, 0.59]	0.34	−0.03	−0.28	0.783	

*Note.* *** *p* < 0.001.

**Table 4:** Correlations between personality structure and parenting dimensions.

Variable	1	2	3	4	5	6	7
1. Personality structure	—						
2. Maternal warmth/support	0.49 ***	—					
3. Paternal warmth/support	0.78 ***	0.64 ***	—				
4. Maternal behavioral control	−0.28 **	−0.29 **	−0.15	—			
5. Paternal behavioral control	−0.56 ***	−0.26 **	−0.50 ***	0.41 ***	—		
6. Maternal psychological control	−0.48 ***	−0.69 ***	−0.50 ***	0.56 ***	0.29 **	—	
7. Paternal psychological control	−0.79 ***	−0.58 ***	−0.82 ***	0.32 **	0.74 ***	0.63 ***	—

*Note*. ** *p* < 0.01, *** *p* < 0.001.

**Table 5:** Stepwise regression predicting personality structure from parenting dimensions.

Model	Variable	*b*	95% CI	*SE_b_*	β	*t*	*p*	*R* ^2^
1	Constant	3.18	[2.96, 3.40]	0.11	—	29.00	<0.001	0.62 ***
	Paternal psychological control	−0.82	[−0.96, −0.68]	0.07	−0.79	−11.82	<0.001	
2	Constant	2.13	[1.56, 2.71]	0.29	—	7.36	<0.001	0.68 ***
	Paternal psychological control	−0.46	[−0.69, −0.24]	0.11	−0.45	−4.13	<0.001	
	Paternal warmth/support	0.41	[0.20, 0.62]	0.11	0.42	3.87	<0.001	

*Note*. *** *p* < 0.001.

**Table 6:** Stepwise regression predicting moral competence from parenting dimensions.

Model	Variable	*b*	95% CI	*SE_b_*	β	*t*	*p*	*R* ^2^
1	Constant	6.18	[1.05, 11.32]	2.58	—	2.40	0.019	0.24 ***
	Paternal warmth/support	8.08	[5.02, 11.15]	1.54	0.49	5.24	<0.001	
2	Constant	17.51	[7.42, 27.60]	5.08	—	3.45	<0.001	0.30 ***
	Paternal warmth/support	5.87	[2.44, 9.31]	1.73	0.36	3.40	<0.001	
	Paternal behavioral control	−4.57	[−8.11, −1.03]	1.78	−0.27	−2.57	0.012	

*Note*. *** *p* < 0.001.

## Data Availability

Due to ethical restrictions and data protection regulations (GDPR), the data supporting the findings of this study are not publicly available. Access may be granted upon reasonable request to the first and corresponding author.

## Appendix A

**Table A1:** Intercorrelation matrix of moral orientations.

Moral Orientation	1	2	3	4	5	6
Typ 1	—					
Typ 2	0.68 **	—				
Typ 3	0.36 **	0.43 **	—			
Typ 4	0.25 *	0.29 **	0.25 *	—		
Typ 5	0.08	−0.02	−0.00	0.62 **	—	
Typ 6	0.12	−0.01	−0.04	0.55 **	0.82 **	—

Note. * *p* < 0.05, ** *p* < 0.01.
